# Classification and clinical behavior of blastic plasmacytoid dendritic cell neoplasms according to their maturation-associated immunophenotypic profile

**DOI:** 10.18632/oncotarget.4146

**Published:** 2015-05-25

**Authors:** Lourdes Martín-Martín, Antonio López, Belén Vidriales, María Dolores Caballero, António Silva Rodrigues, Silvia Inês Ferreira, Margarida Lima, Sérgio Almeida, Berta Valverde, Pilar Martínez, Ana Ferrer, Jorge Candeias, Francisco Ruíz-Cabello, Josefa Marco Buadesa, Amparo Sempere, Neus Villamor, Alberto Orfao, Julia Almeida

**Affiliations:** ^1^ Cancer Research Centre (IBMCC, USAL-CSIC), Institute for Biomedical Research of Salamanca (IBSAL) and Department of Medicine and Cytometry Service, University of Salamanca (USAL), Salamanca, Spain; ^2^ Hematology Department and IBSAL, University Hospital of Salamanca, Salamanca, Spain; ^3^ Hematology Department, Hospital de Santo António dos Capuchos, Lisboa, Portugal; ^4^ Santa Luzia Medical Laboratory, Florianópolis, SC, Brazil; ^5^ Clinical Hematology Department, Hospital de Santo António, Porto, Portugal; ^6^ Hematology Department, Hospital Universidade de Coimbra, Coimbra, Portugal; ^7^ Hematology Department, Hospital Nacional de Niños Dr. Carlos Sáenz Herrera, San José, Costa Rica; ^8^ Hematology Department, Hospital 12 de Octubre, Madrid, Spain; ^9^ Pathology Department, Hospital del Mar, Barcelona. IMIM (Hospital del Mar Medical Research Institute), Barcelona, Spain; ^10^ Immunology Department, Hospital São João, Porto, Portugal; ^11^ Clinical Analysis and Immunology Department, Hospital Virgen de las Nieves, Granada, Spain; ^12^ Hematology Department, Hospital General de Castellón, Castellón, Spain; ^13^ Hematology Department, University Hospital La Fé, Valencia, Spain; ^14^ Hospital Clinic, Barcelona, Spain

**Keywords:** blastic plasmacytoid dendritic cell neoplasm, flow cytometry, maturation profile, acute leukemia, lymphoma

## Abstract

Blastic plasmacytoid dendritic cell neoplasm (BPDCN) is a rare subtype of leukemia/lymphoma, whose diagnosis can be difficult to achieve due to its clinical and biological heterogeneity, as well as its overlapping features with other hematologic malignancies. In this study we investigated whether the association between the maturational stage of tumor cells and the clinico-biological and prognostic features of the disease, based on the analysis of 46 BPDCN cases classified into three maturation-associated subgroups on immunophenotypic grounds. Our results show that blasts from cases with an immature plasmacytoid dendritic cell (pDC) phenotype exhibit an uncommon CD56^−^ phenotype, coexisting with CD34^+^ non-pDC tumor cells, typically in the absence of extramedullary (e.g. skin) disease at presentation. Conversely, patients with a more mature blast cell phenotype more frequently displayed skin/extramedullary involvement and spread into secondary lymphoid tissues. Despite the dismal outcome, acute lymphoblastic leukemia-type therapy (with central nervous system prophylaxis) and/or allogeneic stem cell transplantation appeared to be the only effective therapies. Overall, our findings indicate that the maturational profile of pDC blasts in BPDCN is highly heterogeneous and translates into a wide clinical spectrum -from acute leukemia to mature lymphoma-like behavior-, which may also lead to variable diagnosis and treatment.

## INTRODUCTION

Blastic plasmacytoid dendritic cell neoplasm (BPDCN) is a relatively rare subtype of leukemia/lymphoma - < 1% of hematologic malignancies [1]- which has been recently categorized in the 2008 World Health Organization (WHO) classification of hematological diseases [2] under acute myeloid leukemia (AML) and related precursor neoplasms. Accumulating evidences show that the normal cellular counterpart of BPDCN resides in the plasmacytoid dendritic cell (pDC) lineage [3]. Despite all the above, diagnosis of BPDCN still remains a challenge; this is probably due to several reasons including its overlapping features with other entities, the lack of recurrent and specific chromosomal abnormalities [4, 5], and its heterogeneous clinical presentation with multiple and variable tissue localizations [6–8]. In addition, diagnosis of BPDCN can be difficult to achieve, particularly when blast cells do not completely fit the typical CD4^+^ CD56^+^ HLA-DR^hi^ CD123^+^ lineage (Lin)^−^ immunophenotypic profile [6, 9]. In fact, at present it is now well-established that tumor cells from BPDCN do express markers which have been classically related to other cell lineages such as CD33, TdT, CD79a, CD2 and CD7 [10, 11], and evidences exist about a broad range of atypical phenotypic profiles in a substantial proportion of cases (up to more than one third of cases), including absence of CD56 [12] and CD4 [13]. Such heterogeneity is also observed at the genetic and molecular level, since multiple and diverse, but unspecific, chromosomal and molecular alterations have been identified [4, 5] with even mutually exclusive gene mutations [14]. Therefore, BPDCN are not only clinically heterogeneous but they also show a considerable biologic diversity which in some cases makes diagnosis difficult. This, together with the low prevalence of the disease, has hampered the study of large series of patients, with few exceptions [6, 7, 15], most reports on BPDCN being based on single or just a few cases. Besides, some of the few larger series reported so far are biased, as they generally include only cases showing cutaneous involvement in the absence of systemic disease and/or they have used rather (even retrospective) restrictive phenotypic inclusion criteria; this has probably contributed to the lack of awareness about disease heterogeneity. In such case we might wonder whether or not, the clinical, phenotypical and genetic diversity of BPDCN (e.g. the maturational stage of blast cells and/or their genetic profile) could contribute to further subclassify this entity, similarly to what happens with other neoplasms, such as B and T-cell malignancies or even AML [16].

Here we describe a relatively large series of 46 BPDCN classified into three subgroups, based on the expression of maturation-associated immunophenotypic markers on tumor vs. normal pDC and pDC precursors. Our major goal was to investigate whether the stage of maturational arrest of pDC tumor cells would be associated with distinct and unique clinico-biological and prognostic features of the disease.

## RESULTS

### Clinical and laboratory features at presentation

From all 46 cases analyzed, four (9%) were children (median age of 11 years; range: 8 to 12 years) and 42 were adults (median age of 71 years; range: 25 to 91 years), with a clear male predominance: 35 males (76%) vs. 11 females (24%) (Table 1). Most cases (27/45, 60%) consulted because of organ involvement (e.g. skin lesions) in association with B symptoms (32/45 cases, 71%) (Table 1). In a smaller proportion of cases, diagnosis was made because of bleeding (6/45, 13%), bone pain (4/45, 9%) or just the presence of cytopenias with or without blast cells in a routine blood analysis (5/44, 11%). In line with this, most cases presented with extramedullary involvement (33/45, 73%) mainly consisting of skin lesions (29/45 cases, 64%) and/or, to a lesser extent, testis (6%) and central nervous system (CNS) involvement (9% of cases). Presence of lymphadenopathies, splenomegaly and hepatomegaly was detected on physical examination in 51%, 38% and 22% of cases, respectively. Five patients (16%) had an associated myelodysplastic syndrome (MDS) and 3 (7%) had other non-hematological neoplasms (prostate −*n* = 2- and colon cancer).

**Table 1:** Clinical characteristics of blastic plasmacytoid dendritic cell neoplasms patients classified according to their maturation-associated immunophenotypic profile

	Blast cell phenotype				
Demographics	Total cases	Group 1 (immature)	Group 2 (intermediate)	Group 3 (mature)	*P*-value
	*n* = 46	*n* = 8	*n* = 24	*n* = 14	
Age (years)*	61 ± 22 (8–91)	68 ± 22 (25–91)	56 ± 23 (8–85)	68 ± 19 (11–83)	.05^c^
Children/Adults	4/42 (9%/91%)	0/8 (0%/100%)	3/21 (13%/87%)	1/13 (7%/93%)	NS
Sex (male/female)	35/11 (76%/24%)	6/2 (75%/25%)	15/9 (63%/37%)	14/0 (100%/0%)	.008^c^
**Reason for consulting**					
Routine blood analysis	5/44 (11%)	0/6 (0%)	3/24 (13%)	2/14 (14%)	NS
B & other general symptoms	32/45 (71%)	6/7 (86%)	17/24 (71%)	9/14 (64%)	NS
Bleeding	6/45 (13%)	1/7 (14%)	3/24 (13%)	2/14 (14%)	NS
Bone pain	4/45 (9%)	0/7 (0%)	4/24 (17%)	0/14 (0%)	NS
Organ involvement	27/45 (60%)	0/7 (0%)	14/24 (58%)	13/14 (93%)	≤ .03^a, b, c^
**Physical examination**					
Adenopathies	23/45 (51%)	0/7 (0%)	14/24 (58%)	9/14 (64%)	.007^a, b^
Splenomegaly	17/45 (38%)	0/7 (0%)	13/24 (54%)	4/14 (29%)	.01^a^
Hepatomegaly	10/45 (22%)	0/7 (0%)	7/24 (29%)	3/14 (21%)	NS
Extramedullary involvement	33/45 (73%)	1/7 (14%)	19/24 (79%)	13/14 (93%)	≤ .004^a, b^
- Skin^1^	29/45 (64%)	1/7 (14%)	16/24 (67%)	12/14 (86%)	≤ .02^a, b^
- CNS	4/45 (9%)	0/7 (0%)	3/24 (13%)	1/14 (7%)	NS
- Testis^2^	2/34 (6%)	0/5 (0%)	1/15 (7%)	1/14 (7%)	NS
**Clinical history**					
Associated neoplasia	3/44 (7%)	0/6 (0%)	1/24 (4%)	2/14 (14%)	NS
Myelodysplastic syndrome	5/32 (16%)	2/6 (33%)	1/19 (5%)	2/7 (29%)	NS

Results expressed as number of cases from all cases with available data and (percentage) or as *mean ± one standard deviation (range). Statistically significantly differences were found between ^a^group 1 group 2, ^b^group 1 group 3 and ^c^group 2 group 3. ^1^None showed mucosal involvement, ^2^only in male patients, both children. NS: no statistically significant differences (*p* > 0.05), CNS: central nervous system.

Blood cell counts revealed leukocytosis (> 10 × 10^9^ WBC/l) in 33% of the cases, anemia (< 100g hemoglobin/l) in 63%, thrombocytopenia (< 100 × 10^9^/l) in 64% of the cases and neutropenia (< 1.5 × 10^9^/l) in around half (48%) of cases (Table 2). LDH serum levels were increased in 55% of the patients. The majority of cases (61% and 93%) showed presence of blast cells in both PB (median blast cell percentage of 39%; range: 2% to 83%) and BM (median of 78%; range: 4% to 100%), respectively.

**Table 2:** Blastic plasmacytoid dendritic cell neoplasms classified according to their maturation-associated immunophenotypic profile: laboratory parameters

	Blast cell phenotype				
Laboratory parameters		Total cases	Group 1 (immature)	Group 2 (intermediate)	Group 3 (mature)	*P*-value
		*n* = 46	*n* = 8	*n* = 24	*n* = 14	
Anemia (< 100 g/l)		28/44 (64%)	6/7 (86%)	16/24 (67%)	6/13 (46%)	NS
Leukopenia (< 4 × 10^9^/l)		13/43 (30%)	4/7 (57%)	8/23 (35%)	1/13 (8%)	.03^a^
Neutropenia (< 1.5 × 10^9^/l)		20/41 (49%)	6/7 (86%)	12/22 (55%)	2/12 (17%)	≤ .04^a, b^
Thrombocytopenia (< 100 × 10^9^/l)		28/43 (65%)	3/7 (43%)	16/24 (65%)	9/12 (75%)	NS
Leukocytosis (> 10 × 10^9^/l)		14/43 (33%)	0/7 (0%)	6/23 (26%)	8/13 (62%)	≤ .04^a, b^
Elevated LDH (≥ 450 U/l)		22/40 (55%)	1/6 (17%)	11/21 (52%)	10/13 (77%)	.02^a^
**Presence of blast cells (by morphology)**						
Peripheral blood	% cases	27/44 (61%)	4/7 (57%)	14/24 (58%)	9/13 (69%)	NS
	% blast cells^1^*	39% (2%–83%)	11% (2%–35%)	45% (2%–80%)	39% (10%–83%)	NS
Bone marrow	% cases	41/44 (93%)	8/8 (100%)	21/24 (88%)	12/12 (100%)	NS
	% blast cells^1^*	78% (4%–100%)	68% (46%–73%)	80% (18%–99%)	85% (4%–100%)	NS

Results expressed as number of cases from all cases with available data (percentage) or as *median (range). Statistically significantly differences were found between ^a^group 1 group 3 and ^b^group 2 group 3. ^1^Only in cases with blast cells. NS: no statistically significant differences (*p* > 0.05) found, LDH: lactate dehydrogenase; β_2_-microg: β_2_-microglobulin. No statistical differences were observed among the groups regarding creatinine and C-reactive protein levels.

### Immunophenotypic subgroups of blastic plasmacytoid dendritic cell neoplasms

**Figure 1:**
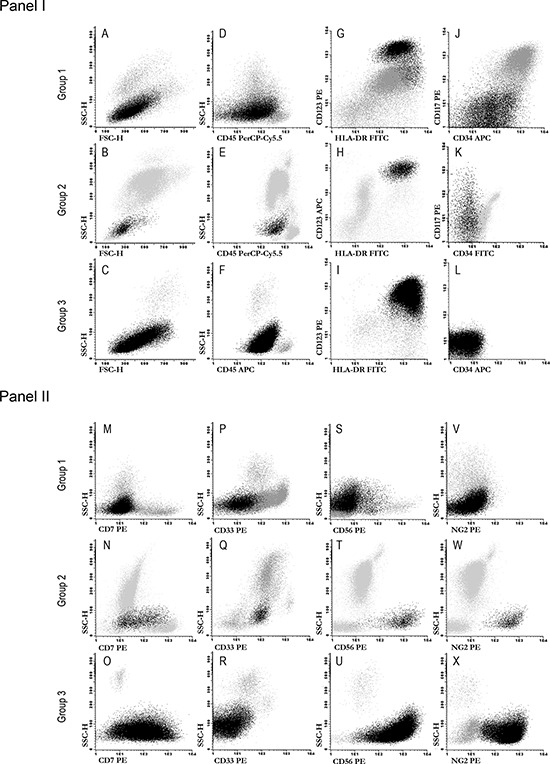
Illustrating dot plots of the three immunophenotypically defined groups of BPDCN The neoplastic plasmacytoid dendritic cell population is depicted as black dots, whereas other leukocytes are depicted in gray. **Panel I:** plots A to I illustrate a combination of markers which are useful for the identification of blastic pDC, while plots J to L show the pattern of expression of CD34 and CD117 for representative cases from the different maturation-associated groups of BPDC neoplasms here defined. **Panel II:** plots M to X display the pattern of expression observed for those markers found to be expressed at statistically different levels among the three maturation-associated groups of BPDC neoplasms.

Patients with BPDCN were divided into three groups according to the maturational stage of the blast/tumor cells as defined by the pattern of expression of CD34 and CD117: 1) immature blastic pDC neoplasms (8/46 cases; 17%) showed expression of CD34 in at least a fraction of the blast cells; 2) intermediate blastic pDC neoplasms (24/46 patients; 52%) typically displayed partial positivity for CD117 in the absence of CD34 expression on the blast cells, and; 3) mature cases (14/46 cases; 30%) had a CD34^−^ CD117^−^ tumor pDC immunophenotype. Of note, among all immature cases (Group 1) neoplastic pDC systematically coexisted with another blast cell population of myeloid or mixed myeloid plus B-lymphoid origin, accounting for a median of 50% of the whole blast cell population (range: 30% to 90%); such coexisting non-pDC lineage blast cells systematically showed strong CD34 and CD117 expression, whereas the pDC component was typically partially positive for CD34 and negative or dim positive for CD117 (Figure 1J, 1K, and 1L). In contrast, both intermediate (Group 2) and mature (Group 3) blastic pDC neoplasms showed no blast/tumor cell population other than the pDC, all these cases being systematically negative for CD34 by definition. Expression of CD117 (in the absence of CD34) was restricted per definition to Group 2, with a median percentage of CD117^+^ cells of 18% (range: 5% to 100%).

**Figure 2:**
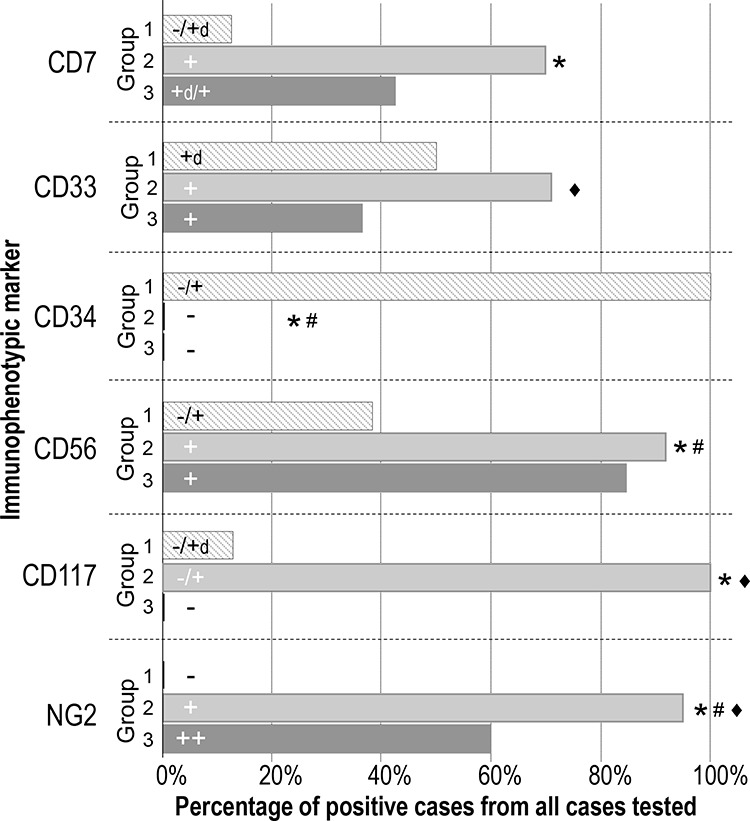
Immunophenotypic differences observed for individual markers on neoplastic pDC from PBDCN patients classified into the three different maturation-associated groups defined in this study Only those markers that showed statistically significant differences -CD7, CD33, CD34, CD56, CD117 and NG2- among the three maturation-associated groups of BPDC neoplasms are shown; the percentage of positive cases and the pattern of expression (intensity) observed for the above listed markers are represented as bars for each group of patients. The correspondence between MFI values and the different categories of intensity of expression defined for the individual markers is shown in Supplementary Table 2. Statistically significant differences were found between *Group 1 vs. Group 2, #Group 1 vs. Group 3 and ♦Group 2 vs. Group 3.

Other immunophenotypic markers which showed different patterns of expression on pDC blast cells included (Supplementary Table 2 and Figure 2): CD7 and CD33 which showed a greater expression among cases in the intermediate immunophenotypic Group 2 (*p* = .008 and *p* = .04, respectively) and to a lesser extent also, in case of CD7, in Group 3 (*p* > .05); CD56, which was dimly expressed only in a fraction of cases in Group 1, but widely and intensely positive on tumor cells from Group 2 and Group 3 cases (*p* ≤ .04), and; NG2, a marker that was absent in Group 1 cases (*p* ≤ .03) but clearly expressed in most of the other more mature Group 2 and Group 3 patients (Figure 1, panel II, plots M to X and Figure 2).

### Clinical and laboratory features of the distinct maturation-associated subgroups of BPDCN

Overall, no significant differences were observed among the three phenotypic groups of BPDCN, as regards the distribution of patients according to age. Although a clear male predominance was found in all groups, this was significantly more pronounced in Group 3, where all 14 cases were males (*p* < .008) (Table 1). Patients in the three groups showed a similar reason for consulting except for cases in Group 1 which less frequently referred because of organ involvement. In line with this, Group 1 cases also showed a significantly lower frequency (vs. the other two groups) of extramedullary involvement (14% vs. 79% and 93%, *p* ≤ .004), lymphadenopathies (0% vs. 58% and 64%, *p* ≤ .007) and splenomegaly (0% vs. 54% and 29%, *p* = .01). Group 2 cases showed intermediate profiles between those of Group 1 and Group 3 patients, as regards the frequency of extramedullary involvement, particularly of skin lesions.

Despite similar levels of BM and PB infiltration by tumor pDCs were found among the three groups, the frequency of anemia, leukopenia and neutropenia progressively decreased from Group 1 to Group 2 and Group 3 cases (Table 2). In contrast, the frequency of thrombocytopenia was slightly higher among the latter two groups. The frequency of cases with increased LDH levels was significantly higher in Group 3 vs. Group 1 cases.

### Patient treatment and outcome

**Figure 3:**
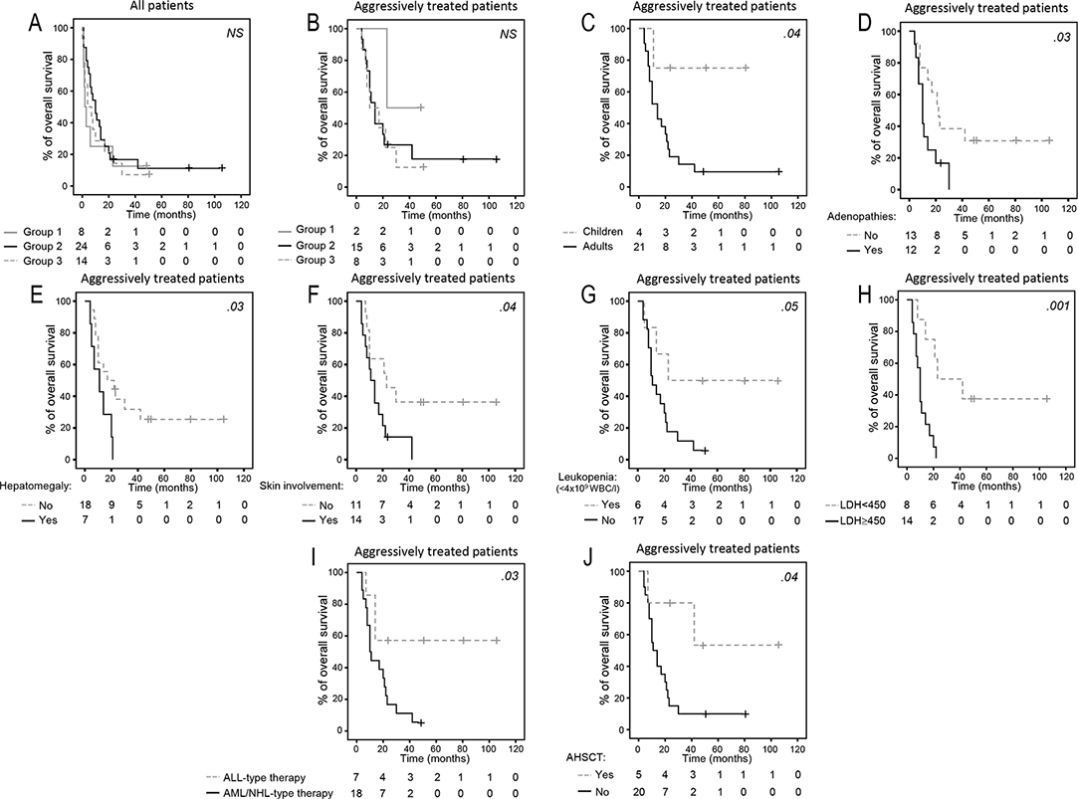
Overall survival of patients with BPDCN Stratified according to the three pDC maturation-associated groups of patients. **Panels A and B,** age at diagnosis **panel C,** presence of lymphadenopathies **panel D,** hepatomegaly **panel E,** skin lesions **panel F**, leukopenia **panel G**, lactate dehydrogenase (LDH) serum levels **panel H,** type of systemic therapy administered –acute lymphoid leukemia (ALL)-type vs. acute myeloid leukemia (AML) or lymphoma (NHL)-type therapies- **panel I,** including allogeneic hematopoietic stem cell transplantation (AHSCT) **panel J.** For all panels but panel A, only those patients who had been treated with an intention to cure, are represented.

All 4 Group 1 patients who were treated with curative intention, systematically received an AML-type regimen; this is in contrast with Group 3 cases, who more frequently received either lymphoma (6/9 cases) or acute lymphoblastic leukemia (ALL; 3/9 patients) directed therapies (*p* = .01) (Table 3). In turn, Group 2 patients received AML (8/19), ALL (5/19) and lymphoma (6/19) associated therapy. Despite the diverse therapies administered, complete remission (CR) was achieved in the great majority of cases (92%) who completed the treatment protocol (*n* = 25), regardless of their phenotypic group (*p* > .05); nevertheless, the outcome was poor with a median overall survival (OS) of only 11 months due to early relapse. Interestingly, relapse and/or progression at the CNS level was observed in a high proportion of cases (33%), mostly among Group 2 and Group 3 (29% and 50%, respectively) patients (Table 3); this was even more frequent when only patients who achieved CR were evaluated (60%), particularly in those cases where a lymphoma-type therapy was administered (Supplementary Table 3). Overall, no significant differences were observed in the outcome of the patients from the three distinct immunophenotypic groups (Table 3 and Figure 3A and 3B). In contrast, adulthood (age at diagnosis > 15 years; *p* = .04) (Figure 3C), presence of lymphadenopathies (*p* = .03) and hepatomegaly (*p* = .03) (Figure 3D and 3E), skin lesions (*p* = .04) (Figure 3F), increased serum LDH (≥ 450U/l; *p* = .001) (Figure 3H) and β_2_-microglobulin (≥ 3 mg/l; *p* = .02) were all associated with a poorer outcome (Table 4). In turn, leukopenia (*p* = .05) (Figure 3G), administration of ALL-type therapy (Figure 3I) and allogeneic stem cell transplantation (Figure 3J) (*p* = .03 and *p* = .04, respectively) were associated with a better outcome. Multivariate analysis of prognostic factors (Table 4) showed that serum LDH levels were the only independent prognostic factor for OS in aggressively-treated patients (*p* = .005, HR = 6.6; 95% CI = 1.8–24.8).

**Table 3:** Type of therapy and clinical outcome of patients with BPDCN classified according to the immunophenotypic-associated maturation profile of blast cells

	Blast cell phenotype				
Type of therapy administered	Total cases	Group 1 (immature)	Group 2 (intermediate)	Group 3 (mature)	*P*- value
	*n* = 46	*n* = 8	*n* = 24	*n* = 14	
AML-type	12/45 (27%)	4/8 (50%)	8/23 (35%)	0/14 (0%)	≤. 01^a, b^
High risk ALL-type	8/45 (18%)	0/8 (0%)	5/23 (22%)	3/14 (21%)	NS
C(H)OP and C(H)OP-like type	12/45 (27%)	0/8 (0%)	6/23 (26%)	6/14 (43%)	.04^a^
AHSC transplantation	5/45 (11%)	1/8 (13%)	4/23 (17%)	0/14 (0%)	NS
Palliative	10/45 (22%)	3/8 (38%)	3/23 (13%)	4/14 (29%)	NS
No treatment	3/45 (7%)	1/8 (13%)	1/23 (4%)	1/14 (7%)	NS
**Clinical outcome**					
Median OS (months)	7 (4–10)	2 (0–4)	10 (5–15)	4 (0–10)	NS
Complete remission^1^	23/25 (92%)	2/2 (100%)	14/15 (93%)	7/8 (88%)	NS
Relapse	17/23 (74%)	1/2 (50%)	10/14 (71%)	6/7 (86%)	NS
CNS relapse/progression	10/30 (33%)	0/3 (0%)	5/17 (29%)	5/10 (50%)	NS
Median OS of aggressively treated non-transplanted cases (months)	11 (5–17)	23^2^	11 (7–15)	10 (0–23)	NS
Overall mortality	42/46 (91%)	7/8 (88%)	22/24 (92%)	13/14 (93%)	NS
Early deaths	12/46 (26%)	4/8 (50%)	4/24 (17%)	4/14 (29%)	NS
Deaths after remission	18/23 (78%)	1/2 (50%)	11/14 (79%)	6/7 (86%)	NS

Results expressed as number of cases from all cases with available data (percentage). Statistically significantly differences were found between ^a^group 1 group 3 and ^b^group 2 versus group 3. ^1^Only in aggressively treated patients, ^2^only one case. BPDC: blastic plasmacytoid dendritic cell; AML: acute myeloblastic leukemia; ALL: acute lymphoblastic leukemia; NS: no statistically significant differences (*p* > 0.05); AHSC: allogeneic hematopoietic stem cell; OS: overall survival; CNS: central nervous system.

**Table 4:** Prognostic factors (univariate and multivariate analysis) for overall survival among aggressively-treated patients with blastic plasmacytoid dendritic cell neoplasms (*n* = 25)

Patient features		% of cases	Median (months)	Uni *P*- value	Multi *P*- value	Hazard Ratio	95% CI
Age	≤ 15 y	16	NR	.04			
	> 15 y	84	14				
Adenopathies	No	52	22	.03			
	Yes	48	10				
Hepatomegaly	No	72	17	.03			
	Yes	28	11				
Skin involvement	No	44	23	.04			
	Yes	56	11				
Leukopenia (< 4 × 10^9^/l)	No	74	11	.05			
	Yes	26	23				
Elevated LDH (≥ 450U/l)	No	36	23	.001	.005	6.6	1.8 – 24.8
	Yes	64	10				
Increased β_2_-M (≥ 3 mg/l)	No	43	42	.02			
	Yes	57	10				
ALL-type treatment	No	28	10	.03			
	Yes	72	NR				
AHSC transplantation	No	80	11	.04			
	Yes	20	NR				
Relapse	No	32	NR	.02			
	Yes	68	14				

Uni: univariate analysis, Multi: multivariate analysis, CI: confidence interval, NR: not reached, LDH: lactate dehydrogenase, β_2_-M: β_2_-microglobulin, ALL: acute lymphoblastic leukemia, AHSC: allogeneic hematopoietic stem cell.

## DISCUSSION

BPDCN is a rare but very aggressive disease whose nomenclature, ontogeny and underlying biology have evolved over the years, since its first description by Brody et al. in 1995 [17]. Despite the number of cases reported in recent years has increased, as well as our knowledge about the biology of its postulated normal cellular counterpart (i.e. pDC lineage cells) [9, 18–20], the diagnosis of the disease still remains a challenge. This is due in part, to the clinical and phenotypical diversity observed among the patients and the overlapping features with other hematologic malignancies. In order to elucidate whether the clinical heterogeneity of BPDCN is related to the maturation stage of pDC tumor cells, here we analyzed a relatively large series of 46 patients, whose general clinical features were fully compatible with those previously reported for this rare entity [6, 7], except for the proportion of cases presenting with skin involvement that was lower (64% vs. 76%–100%) in our vs. other series [6, 7, 15, 21]. Although there is no definitive explanation for such difference, it might be due to the fact that until recently, skin lesions have been considered one of the major features that point to the diagnosis of BPDCN and consequently, a fraction of BPDCN patients, who have no cutaneous manifestations, might have gone undetected/not identified. Alternatively, a bias in sample selection could also exist, as more blood and BM vs. tissue samples from complex cases are referred from other centers to our institution.

Once we grouped our cases according to the immunophenotypic maturation profile of pDC-lineage tumor cells, important differences were noted between the immature and the more mature cases, the intermediate group showing also intermediate features. Despite CD34 has been frequently considered as an exclusion marker for the diagnosis of BPDCN [11, 22], this marker was expressed in a fraction of pDC-lineage commited blast cells in around one fifth of our cases. Interestingly, these CD34^+^ BPDCN cases systematically had a second CD34^+^ blast cell population with a minimally differentiated (non-lymphoid) phenotype. These results support and extend on previous observations by the Hellenic Dendritic Cell Leukemia Study Group, which found similar phenotypic and clinical characteristics to those of our Group 1 cases in 4/26 (15%) of their BPDCN patients [6]. If the WHO 2008 criteria [2] would be strictly applied, these cases would be classified as acute leukemia of ambiguous or minimally differentiated myeloid lineage, because of the lack of clear differentiation markers for cell lineages other than pDC. Of note, lymphoid lineage-committed blast cells have also been reported in a smaller proportion of these CD34^+^ cases ([6] and personal unpublished observations). Previous studies have shown that among CD34^+^ pDC cases, both the pDC lineage-committed and the minimally differentiated blast cell population coexisting in individual patients, share the same chromosomal aberrations [23, 24], which suggests that both cell populations arise from a common ancestral leukemia cell, and that the pDC-lineage component may represent the more differentiated blast cell compartment. Of note, in these CD34^+^ cases, the pDC-lineage committed blast cell population frequently did not show aberrant expression of either CD56 or NG2 [6, 23], in contrast to most other more mature (Group 2 and Group 3) BPDCN cases. Furthermore, this subset of CD34^+^ BPDCN cases also showed a unique clinical behavior vs. the other cases; e.g. at presentation they typically lacked skin lesions and extramedullary disease. These results indicate that in these patients blast cells appear to be almost exclusively confined to the BM with some spreading to PB, reflecting a typical “acute leukemia-like” behavior, associated in most cases with multiple cytopenias. This is in line with the immunophenotypic profile of blast cells from cases classified as immature (Group 1) BPDCN, supporting the notion that they would mimic a stem cell malignancy whose blast cells would have limited ability to differentiate into the early stages of pDC lineage. Conversely, patients classified under Group 3, displayed a more mature pDC immunophenotype, associated with a greater frequency of extramedullar involvement (particularly in the skin) and spread into the lymph nodes and other secondary lymphoid tissues; such clinical behavior mimics that of aggressive lymphomas. Altogether, these results point out to a more mature and differentiated tumor pDC, blocked at a stage where the pDC precursor already leaves the BM via PB, with a greater migration capacity into peripheral tissues and secondary lymphoid tissues, which translates into frequent extramedullary organ involvement. In line with this hypothesis, Group 2 cases showed an intermediate phenotypic and clinical behavior between the other two groups (immature and mature) of BPDCN patients. These results support the notion that BPDCN originates from a BM cell precursor that secondarily involves the skin [25] and other peripheral tissues and lymphoid organs, at least in the great majority of cases.

Despite the here defined maturation-associated subgroups of BPDCN displayed a clearly different clinical behavior at presentation, no significant differences were observed among them as regards disease outcome, with systematically low (i.e. less than a year) OS rates. However, it should be noted that different treatments were given to these patients due to their heterogeneous clinical presentation and equivocal tumor cell features at presentation. Of note, high CR rates were obtained, regardless of the type of therapy administered. However, most cases showed an aggressive clinical course with early recurrence of the disease, resistance to therapy and a dismal prognosis with median overall survival rates of less than a year, fully in line with previous observations [25]. Despite the above, more detailed analysis of patient outcome according to therapy showed that cases treated with ALL-type protocols had a better outcome than the other patients, particularly than those who received lymphoma-type regimens (Supplementary Table 3); these results are also in line with previous observations [6, 21], although younger patients (e.g. children) were mostly included in the group treated with ALL-like protocols. Therefore, the question remains about whether the treatment itself or age at diagnosis, would explain the apparently better prognosis of this subgroup of BPDCN patients. In this regard, it should also be emphasized that the only two adults who survived so far, were both treated with an allogeneic stem cell transplant following ALL-type therapy in one case and AML-type chemotherapy in the other patient. Despite the low number of cases included in each group, our results would reinforce previous observations suggesting a better outcome for patients treated with ALL-type and/or allogeneic stem cell transplantation [6, 7, 21, 26].

Although the potential impact of age cannot be ruled out, we may speculate that the better outcome of cases treated with ALL-type chemotherapy could be due to systematic administration of CNS prophylaxis, since a high percentage of the cases (60%) showed CNS involvement at relapse, particularly among non-ALL-type chemotherapy treated patients (all but one, who received an ALL-type protocol). Despite the broad variety of prognostic factors -including therapy- found to be associated with patient outcome, LDH emerged as the only independent prognostic factor for aggressively-treated patients, in line with the strong predictive value of LDH in other hematological malignancies, particularly mature/peripheral lymphoid tumors [27, 28].

In summary, here we show that tumor cells from BPDCN may show a highly variable maturational profile, which translates into a heterogeneous clinical behavior ranging from that of acute leukemia to peripheral/mature lymphomas, such wide clinical spectrum potentially leading also to variable differential diagnoses and administration of distinct treatment modalities. Despite the dismal prognosis of the great majority of patients, usage of ALL-type therapy with CNS prophylaxis and/or allogeneic stem cell transplantation appears to be, among all regimens administered, the only effective ones. Overall, these results support the unique clinical and prognostic behavior of this subgroup of non-lymphoid neoplasms, which requires redefined diagnostic criteria and improved/novel [29] treatment strategies/regimens (e.g. CNS prophylaxis and consolidation regimens) for prolonged CR and survival rates.

## MATERIALS AND METHODS

### Patients and samples

A total of 46 patients (4 children and 42 adults; 35 males and 11 females) diagnosed with BPDCN (according to WHO 2008 criteria [2]) and who were referred to the Cytometry Service of the Cancer Research Centre of the University of Salamanca (Salamanca, Spain), were included in this study. Diagnosis was prospectively made, based on co-expression of HLA-DR^++^ and CD123^+/++^ (which strongly supports a plasmacytoid dendritic cell origin [11, 30]) together with CD4 positivity, dim expression of CD45 and intermediate forward scatter (FSC)/sideward scatter (SSC) values, after blast cell commitment to other myeloid and lymphoid cell lineages had been excluded by morphological, immunophenotypical and molecular analyses (e.g. germline TCR and IGH gene configurations). The vast majority of cases also showed expression of CD36, further supporting a BPDCN diagnosis in the presence of the above referred phenotype.

In every patient, bone marrow (BM), peripheral blood (PB) and/or fine-needle aspiration (FNA) tissue samples were obtained and studied at diagnosis, before any therapy had been administered. Clinical and laboratory data was retrospectively collected from the referring centers. At the moment of closing the study, median follow-up was of 7 months (range: < 1 to 104 months), 41 patients had died and 5 remained alive. The study was approved by the Ethics Committee of the Cancer Research Center, and performed following the Declaration of Helsinki. Each participant gave his informed consent prior entering the study.

Systemic therapy was administered to 32/46 patients and consisted of standard AML-type (cytarabine in combination with idarubicin, mitoxantrone or daunorubicin), acute lymphoid leukemia (ALL)-type (Spanish PETHEMA LAL-AR/2003 protocol and hyper-CVAD) [31] or lymphoma-type (i.e. C(H)OP) protocols, according to local criteria at the referring centers. The remaining patients received only palliative care based on local criteria.

### Flow cytometry immunophenotyping

Multiparameter flow cytometry immunophenotypic studies were performed on whole BM, PB and FNA samples obtained from lymphoid tissues and/or skin lesions, using multicolor [3, 4, 6–8, 11] combinations of a large panel of monoclonal antibodies (Supplementary Table 1); since 2009 these included the EuroFlow panels [32]. In all cases, samples were stained using a direct immunofluorescence technique, as described elsewhere [20]. For data acquisition, a FACSCalibur and FACSCanto II flow cytometers (Becton Dickinson Biosciences –BD-, San Jose, CA, USA) were used. The Infinicyt (Cytognos, Salamanca, Spain) software program was used for data analysis.

### Cytomorphological and histopathological analyses

Cytological analysis of May-Grünwald-Giemsa stained PB and BM smears was performed by conventional microscopy. In turn, conventional histopathological analyses were also performed in parallel for those cases showing lymph node and/or skin involvement, to further confirm tissue infiltration by pDC blast cells.

### Statistical methods

Conventional descriptive and comparative statistics – Mann-Whitney U non-parametric test (for continuous variables) and the χ^2^ test (for categorical variables)- were calculated using the PASW 19 program (IBM SPSS Statistics, IBM, Armonk, NY, USA). Overall survival curves (OS) were plotted according to the Kaplan-Meier method and compared using the (one-sided) log-rank test. Multivariate Cox proportional hazard models (stepwise regression) were applied to explore the independent effect of each parameter under study (including immunophenotypical BPDCN subgrouping) on OS. Hazard ratio and 95% confidence interval were also estimated. *P* values < 0.05 were considered to be associated with statistical significance.

## SUPPLEMENTARY TABLES






