# Supplementary material for: Classification and clinical behavior of blastic plasmacytoid dendritic cell neoplasms according to their maturation-associated immunophenotypic profile
Source: Oncotarget. 2015 May 25;6(22):19204–16. doi: 10.18632/oncotarget.4146 (PMC4662485; doi:10.18632/oncotarget.4146)
Supplement: Supplementary file 1 [file oncotarget-06-19204-s001.pdf]

SUPPLEMENTARY TABLES

Supplementary Table S1. Monoclonal antibody reagents used for the immunophenotypic characterization of blastic plasmacytoid dendritic cell neoplasms

Supplementary Table S2. Immunophenotypic characteristics of blastic plasmactyoid dendritic cell neoplasms according to the three phenotypic-based groups defined in this study

Supplementary Table S3. Clinical features and outcome of aggressively-treated blastic plasmacytoid dendritic cell neoplasms (*n* = 25) according to the type of polychemotherapy regimen received

Patients features	ALL-type *n* = 7	AML-type *n* = 9	C(H)OP-type *n* = 9	*P*-value
Age (years)	36 ± 27 (8–70)	47 ± 17 (10–65)	67 ± 15 (42–81)	≤.03[a, b]
Age distribution (child/adult)	3/4 (43%/57%)	1/8 (11%/89%)	0/9 (0%/100%)	NS
Sex (male/female)	6/1 (86%/14%)	3/6 (33%/67%)	8/1 (89%/11%)	.03[b]
**Clinical outcome**				
Median EFS (months)	NR	8 (7–9)	7 (4–10)	.01[a]
Median OS (months)	NR	11 (8–14)	10 (4–16)	.03[a]
Complete remission	7/7 (100%)	9/9 (100%)	7/9 (78%)	NS
Relapse	3/7 (43%)	7/9 (78%)	7/7 (100%)	.04[a]
CNS relapse/progression	1/6 (17%)	1/4 (25%)	5/6 (83%)	.04[a]
AHSC transplantation	2/7 (29%)	3/9 (33%)	0/9 (0%)	NS
Overall mortality	3/7 (43%)	8/9 (89%)	9/9 (100%)	.02[a]
Deaths after remission	3/7 (43%)	8/9 (89%)	7/7 (100%)	.04[a]
Deaths after transplant	0/2 (0%)	2/3 (67%)	NA	NS

Results expressed as number of cases from all cases with available data (percentage) except for age, where mean ± one standard deviation is used, and EFS and OS where results are expressed as median and lower and upper limits of 95% confidence interval. [a]ALL-type therapy vs C(H)OP-type therapy [b]AML-type therapy vs C(H)OP-type therapy. BPDC: blastic plasmacytoid dendritic cell; ALL: acute lymphoblastic leukemia; AML: acute myeloblastic leukemia; C(H)OP-like therapy: COP/CHOP-like therapy; NS: no statistically significant differences ($p > 0.05$); EFS: event free survival; NR: not reached; OS: overall survival; CNS: central nervous system; AHSC: allogeneic hematopoietic stem cell; NA: not applicable.
